# Massive parallel *IGHV* gene sequencing reveals a germinal center pathway in origins of human multiple myeloma

**DOI:** 10.18632/oncotarget.3644

**Published:** 2015-04-10

**Authors:** Graeme Cowan, Nicola J. Weston-Bell, Dean Bryant, Anja Seckinger, Dirk Hose, Niklas Zojer, Surinder S. Sahota

**Affiliations:** ^1^ Institute of Immunology and Infection Research, Centre for Immunity, Infection and Evolution, University of Edinburgh, UK; ^2^ Tumour Immunogenetics Group, Cancer Sciences Academic Unit, Faculty of Medicine, University of Southampton, UK; ^3^ Medizinische Klinik V, Universitätsklinikum Heidelberg, Heidelberg, Germany; ^4^ Center for Oncology and Hematology, 1st Department of Medicine, Wilhelminenspital, Vienna, Austria

**Keywords:** multiple myeloma, pathogenesis, IGHV genes, germinal center

## Abstract

Human multiple myeloma (MM) is characterized by accumulation of malignant terminally differentiated plasma cells (PCs) in the bone marrow (BM), raising the question when during maturation neoplastic transformation begins. Immunoglobulin *IGHV* genes carry imprints of clonal tumor history, delineating somatic hypermutation (SHM) events that generally occur in the germinal center (GC). Here, we examine MM-derived *IGHV* genes using massive parallel deep sequencing, comparing them with profiles in normal BM PCs. In 4/4 presentation IgG MM, monoclonal tumor-derived *IGHV* sequences revealed significant evidence for intraclonal variation (ICV) in mutation patterns. *IGHV* sequences of 2/2 normal PC IgG populations revealed dominant oligoclonal expansions, each expansion also displaying mutational ICV. Clonal expansions in MM and in normal BM PCs reveal common *IGHV* features. In such MM, the data fit a model of tumor origins in which neoplastic transformation is initiated in a GC B-cell committed to terminal differentiation but still targeted by on-going SHM. Strikingly, the data parallel *IGHV* clonal sequences in some monoclonal gammopathy of undetermined significance (MGUS) known to display on-going SHM imprints. Since MGUS generally precedes MM, these data suggest origins of MGUS and MM with *IGHV* gene mutational ICV from the same GC B-cell, arising via a distinctive pathway.

## INTRODUCTION

In multiple myeloma (MM), malignant plasma cells accrue in the bone marrow (BM) expressing CD138, a marker of terminal B-cell differentiation. In MM, an early question has been the nature of the cell of origin or normal B-cell counterpart in which neoplastic transformation begins. Addressing this question, immunoglobulin variable gene (*IGV*) analyses in MM have provided pivotal insights.

*IGV* genes encode the antigen-binding domain of the immunoglobulin molecules in the B-cell receptor (BCR), a receptor that is essential to survival and maturation of normal B-cells [1]. Pathways of B-cell maturation are determined by the context in which antigen is seen via the BCR. In the presence of cognate T-cell help, a distinctive phase of differentiation is initiated in the germinal center (GC), in secondary follicles of lymphoid organs that leads to the mainstay of memory, generating memory B-cells or long-lived plasma cells (LLPCs) located in the BM [2, 3]. The GC can be morphologically compartmentalized to two areas, a dark zone (DZ) of proliferating B-cells or centroblasts (CBs) that downmodulate BCR and express CXCR4, and the light zone (LZ) of small B-cells or centrocytes (CCs) expressing BCR and CXCR5 [4]. Affinity maturation to improve BCR fit for antigen occurs by targeted somatic hypermutation (SHM) of *IGV* genes, initiated by activation induced cytidine deaminase (AID) that is expressed at high levels in CBs in the DZ [2–6]. Following proliferation, selection of B-cells by antigen occurs after migration to the LZ in CCs, and is dependent on affinity for antigen complexes presented by follicular dendritic cells and interaction with follicular T_H_ (T_FH_) cells [7]. Precise imaging experiments have shown that CBs and CCs shunt bi-directionally between the DZ and LZ [8], to permit continued induction of SHM. Isotype class switch recombination (CSR) also occurs in the GC, and can be initiated in cells on the cusp of proliferating clonally [9]. CSR is an irrevocable process of deletional DNA recombination events dependent on AID activity [6]. B-cells selected by antigen exit the GC to two fates, circulating as CD27^+^ human memory B-cells or as cells committed to LLPC maturation that home and reside in the BM [3–5, 10, 11].

*IGV* gene analysis in B-cell neoplasms can delineate transit via the GC, and the precise pattern of SHM in clonally-related transcripts provides clear insight into when neoplastic transformation is likely to have occurred [12]. In MM, early data from heavy chain *IGHV* gene analyses from our group and others revealed an extensive SHM load in tumor-derived sequences, and sequencing of tumor transcripts segregated by standard cloning strategies and Sanger sequencing revealed intraclonally homogeneous *IGHV* sequences, consistent with transformation occurring at a post-follicular stage with SHM silenced [12]. However, when we examined *IGHV* gene sequences in monoclonal gammopathy of undetermined significance (MGUS), we observed that tumor-derived *IGHV* sequences revealed a marked intraclonal variation (ICV) in mutation patterns in some cases, implicating neoplastic arrest in MGUS with ICV at an earlier stage of maturation, at a stage consistent with exposure of the cell of origin to on-going SHM in the GC [13]. Furthermore, when we examined progression of MGUS-MM in paired cases by *IGHV* analysis, we observed that in one MGUS-MM pair, we could locate variant MGUS-like *IGHV* mutations also in MM clonal sequences [14]. At that point in our studies, we suggested that MM evolves by cloning out of MGUS-like cells [14]. Given the technical limitations of cloning and sequencing strategies used earlier in the analysis of the MM clone by *IGHV* analysis, and our initial observations that clonal variants could persist in MM from an MGUS stage, we reasoned that if the *IGHV* sequencing depth is ramped up, as currently feasible by next generation sequencing, it may be possible to observe ICV more commonly in MM, with new implications for tumor origins. To do this, we have carried out massive deep parallel sequencing of *IGHV* genes in 4 of 4 symptomatic IgG MM cases at presentation, comparing findings with 2 of 2 cases of normal PCs purified from BM and 1 control IgA MM case in which non-tumor IgG transcripts were amplified. Our data substantiate our hypothesis, and we find clear evidence for ICV among clonal MM-derived *IGHV* sequences. This suggests as the most likely model that MM can originate in a GC B-cell committed to terminal maturation but still targeted by SHM.

## RESULTS

### Deep sequencing *IGHV* gene IgG transcripts and error rate

*IGHV* gene IgG transcripts from malignant and normal BM PCs were analyzed by next-generation deep sequencing. Following read-pair alignment and sequence quality filtering, the total number of high quality reads for both technical replicates in each donor ranged from 97,698 to 2,009,338. The percentage of reads for each unique *IGHV* sequence was congruent between the two technical replicates (Figure S1). To increase confidence that reads represented true sequence variants rather than errors introduced by base mis-incorporation during PCR or sequencing mis-reads, sequence variants were only analyzed where they were present at high frequency in both technical replicates. No clonotype sequences with high read counts were exclusive to a single technical replicate.

### *IGHV* gene use in MM

**Figure 1:**
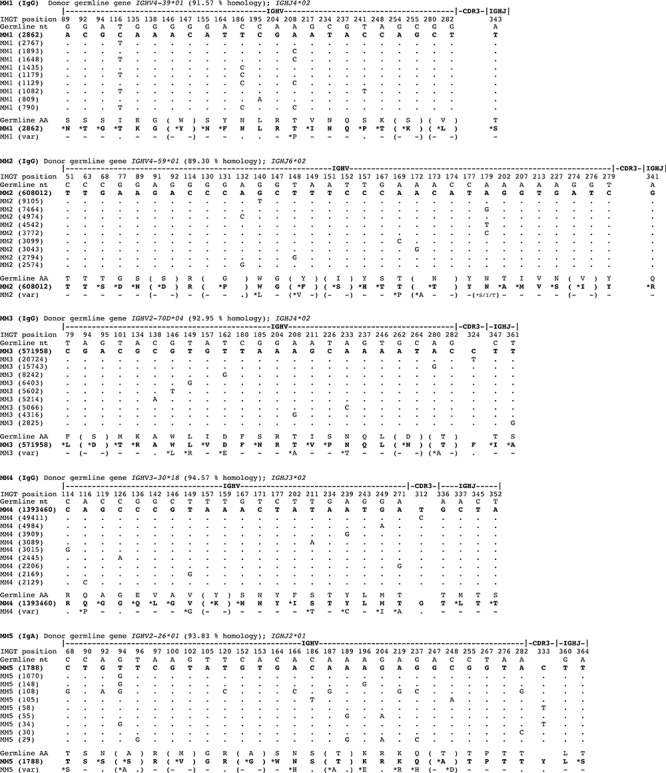
Marked intraclonal variation in tumor-derived *IGHV* gene sequences in MM The pool of IgG transcripts identified from deep sequencing were aligned by computational analysis with donor germline *IGHV* genes and CDR3 identity used to delineate tumor-derived clonal sequences. Only selected informative codons are shown, focusing on revealing replacement nucleotides that arise by SHM for purposes of clarity. The % homology to germline *IGHV* gene of dominant clonal sequence in MM1-4 is stated in each case, and the dominant sequence is shown aligned to the germline gene. Data on the top 10 clonally-derived MM tumor *IGHV* sequences are shown aligned to the dominant sequence or top read, and not the germline gene to reveal the significant intraclonal variation observed in mutation patterns (dots denote sequence identity to the top read). For MM1 and MM2 aligned sequence matches start at position 43, for MM3 at position 65, and for MM4 at position 68. Translation to deduced amino acid (aa) sequences are shown aligning germline aa with replacement aa resulting from mutations in tumor *IGHV* gene sequences (denoted with *, and no change as -). No dominant clonal *IGHV* gene sequence was identified in the control MM5.

Single tumor-derived clonal *IGHV* sequences were readily identified in 4 of 4 MM cases with identical CDR3 nucleotide motifs to establish monoclonality, and utilized germline genes IGHV2-70D*04, IGHV3-30*18, IGHV4-39*01, and IGHV4-59*01 respectively, each displaying marked imprint of SHM with % homology to germline varying between 89.3 – 94.6 (Figure 1). In these samples, the same CDR3 sequence was found in between 74.0 – 87.3% of reads, surpassing cut-offs of >5% used to identify clonal *IGHV* gene use by deep sequencing [15]. Total reads from 2 replicates with identical CDR3 in respective cases were: MM1 (1,660,375); MM2 (1,232,656); MM3 (1,218,020); and MM4 (2,009,338) (Table S1). No dominant clonal IgG sequence was identifiable in control MM5 case, as expected.

### Pattern of SHM in MM-derived *IGHV* gene sequences

Analysis of all reads sharing clonal CDR3 motifs revealed subclonal variant sequence expansions in monoclonal *IGHV* gene tumor-derived sequences in MM1-4. Multiple variant sub-clonal sequence expansions were identified. Of these, the top 10 subclonal variant sequences are shown, each aligned to the dominant sequence at the nucleotide level, with the top read shown aligned to germline (Figure 1). The number of sequences identified in the top 10 tumor-derived subclonal expansions differed: MM1 (790–2,862 sequences); MM2 (2,574–608,012 sequences); MM3 (2825–571,958 sequences); and MM4 (2129–1,393,460 sequences) (Figure 1).

Tumor-derived subclonal sequences as shown were identical, e.g. in MM1 the dominant subclonal sequence gave a count of 2862 and these were all homogeneous in sequence (Figure 1). In MM1, the dominant subclonal homogeneous sequence and the second-most prevailing variant sequence were comparable in number. In 3 of 4 MM cases (MM2-4), the dominant subclonal sequence far surpassed in number the second-most predominant variant sequence. These data indicate that the level of ICV differed between the 4 MM cases and that in cases MM2-4 the tumor clones are dominated by a homogeneous clonal outgrowth.

Nevertheless, the proportion of variant sub-clonal sequence reads over and above the dominant sequence or top read were substantial and were found to comprise 99.8% in MM1, 50.7% in MM2, 53.0% in MM3 and in MM4 30.7% of the entire tumor clone (calculated from reads in Table S1).

Strikingly, in each of the 4 MM cases, there is extensive evidence of ICV as revealed by multiple variant *IGHV* sequences derived from a single tumor clone (Figure 1). These data derive from 4 ‘biological replicates’ (MM1-4) in experimental terms and yield reproducible results.

### Profile of non-tumor PCs in MM samples

**Figure 2:**
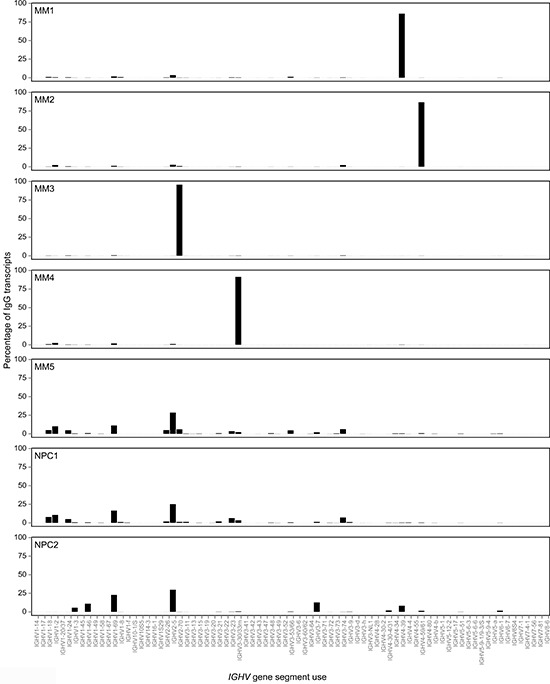
Monoclonal tumor-derived *IGHV* gene sequences dominate the compendium of IgG transcripts in MM as compared with oligoclonal expansions of *IGHV* gene sequences derived from non-tumor or normal plasma cells in the bone marrow All IgG transcripts identified by deep sequencing were aligned with germline *IGHV* gene databases and CDR3 identity used to group clonally related *IGHV* sequences in MM1-4, NPC1-2 and MM5. The frequency of each dominant clone in MM and oligoclonal expanions in normal plasma cells or non-tumor plasma cells are shown as a percentage of the total number of distinct IgG transcript reads per sample, visualized as a bar chart.

IgG transcript profiles in MM1-4 indicated that profiles of non-tumor IgG PCs were markedly depressed (Figure 2).

### *IGHV* gene use in normal PCs

A diverse repertoire of *IGHV* gene use was observed in IgG transcripts in normal PCs in samples NPC1-2; however, most of these were expressed at low frequencies (data not shown). IgG transcripts were grouped by *IGHV* gene germline use and expression levels mapped on a linear scale based on % frequencies (Figure 2). The overall use of *IGHV2-5* germline gene was the largest and common to NPC1-2 and MM5, followed by frequency of usage of the *IGHV1-69* gene in these 3 samples (Figure 2). In the control IgA MM5 case, usage of germline *IGHV* gene elements resembled that seen in NPC1-2 (Figure 2).

Oligoclonal expansions were however extensively observed in NPC1-2 based on identical CDR3, and the largest expansion in NPC1 accounted for 2.4% of all IgG transcripts and in NPC2 for 7.8%. Our data also indicate that in deep sequencing of IgG transcripts in MM, the cut-off required to identify the tumor clone is likely to be higher (>7.8%).

### Pattern of SHM in clonally related *IGHV* gene sequences in normal PCs

**Figure 3:**
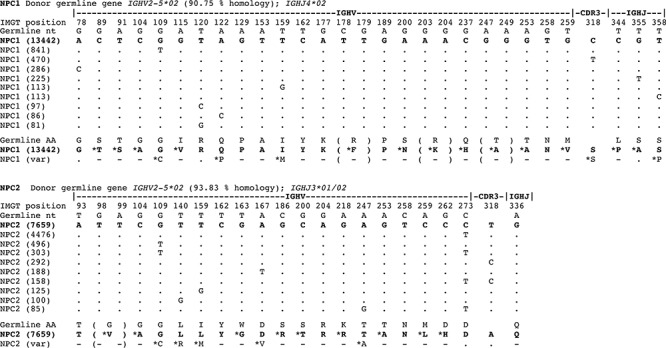
Oligoclonal expansions identified in *IGHV* gene sequences derived from normal plasma cells in the bone marrow reveal marked intraclonal variation IgG transcript profiles obtained by deep sequencing were aligned with germline *IGHV* genes and CDR3 identity used to identify oligoclonal expansions in normal human plasma cells. Only informative selected codons are shown, not entire *IGHV* genes. The largest oligoclonal expansion in each of NPC1 and NPC2 samples are shown, indicating % homology to donor germline gene, and the top 10 clonally-related transcripts are aligned at the nucleotide level to show sequence identity to the top read (by dots) or replacement nucleotides as shown in sequences. The data reveals a significant level of intraclonal variation in oligoclonal expansions of normal plasma cells in *IGHV* gene sequence mutations. Translation to deduced amino acid (aa) are also shown comparing germline with plasma cell *IGHV* gene sequence. For both NPC1 and NPC2 aligned sequence matches start at position 65.

**Figure 4:**
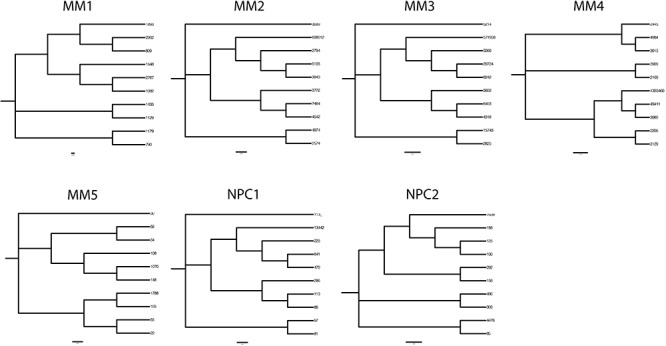
Phylogenetic trees delineating hierarchical derivation of clonally related *IGHV* gene sequences in MM and in normal plasma cells in the bone marrow Phylogenetic analysis of the 10 most prevalent clonal expansions linked by identical CDR3 motifs in MM and normal plasma cells were aligned using Clustal Omega and nearest neighbor joining trees plotted. Node numbers indicate read count for each sequence variant.

The largest oligoclonal expansions of IgG transcripts in NPC1-2 were evaluated, and subclonal variants related by identical CDR3 in each expansion aligned at the nucleotide level, using the top 10 most prevalent variant sequences (Figure 3). In 2 of 2 normal BM PC populations, oligoclonal expansions reveal marked ICV (Figure 3), comparable in mutational load and variation to that observed in MM1-4. Analysis of phylogenetic trees indicated comparable degrees of branching and therefore exposure to comparable rounds of SHM in evolution of clonally-related normal PCs and of clonally-related malignant PCs (Figure 4).

## DISCUSSION

On-going SHM occurs in B-cells during extensive proliferation within the DZ of a GC, whereas in the LZ key selection events dictate B-cell fate [1, 4, 5]. Improved affinity for antigen by SHM allows preferential access to T_FH_ cells in the LZ for extended stimuli, which appear to dictate either re-entry to the DZ or differentiation [7, 16]. The precise nature of signals and immunological synapses that determine fate as memory B-cell or PC in the GC however are as yet not fully defined, but are orchestrated by specific transcription factors. Of these, IRF4 is required for induction of AID and CSR and for derivation of PCs [17]. Specifically, graded expression of IRF4 regulates AID levels and induction of Blimp-1, an obligate requirement for terminal differentiation of PCs [18]. Interestingly, a ‘pre-PC’ stage has been described that delineates GC B-cell commitment to PC fate and precedes Blimp-1 expression and is able to undergo SHM, also expressing Flt3 and Embigin that are repressed by Pax5 at an earlier stage [19]. Normal B-cells that undergo SHM but fail to improve fit for antigen undergo apoptosis by a default mechanism [2, 20, 21]. This check-point however may be by-passed by transformation, as suggested by GC B-cells that carry the t(14;18) translocation in follicular lymphoma [22].

The clonal nature of PCs derived from selection events in the GC can be gauged from *IGHV* gene analysis in data from the T-dependent response to a (4-hydroxy-3-nitrophenyl) acetyl-protein conjugate antigen in murine B-cells, seeking to assess a role for affinity in antibody secreting cell (ASC) selection and migration to the BM [23]. These experiments clearly show clonal waves of isotype switched antigen specific ASCs migrating to the BM, displaying clonally-related V_H_186.2 gene sequences with marked ICV. These observations also indicate that clonal variants that are antigen-specific are selected in the GC to mature sequentially or synchronously to ASCs for homing to the BM, with late GC emigres displaying further evidence of accrual of SHM imprints in V_H_186.2 sequences [23], consistent with re-entry to DZ of antigen-selected B-cells.

Data from our analysis of *IGHV* genes in normal human PCs from the BM reflect these features of selection in antigen-specific murine ASCs [23]. We observed oligoclonal expansions of normal PCs with marked ICV in each. Comparable observations were also reported in a study of normal BM PCs from a single donor by deep sequencing, with the largest *IGHV3-7* derived clonal expansion providing evidence of ICV to a level comparable to that seen in our data on normal PCs [24]. These findings delineate that LLPCs in the BM comprise multiple variant subclones within oligoclonal expansions to specific antigens, which persist over time at this site. Normal LLPCs in the BM are known to derive mainly from GC B-cells [11, 25].

In our earlier study of MGUS, *IGHV* gene analysis carried out by conventional cloning and sequencing revealed monoclonal tumor-derived sequences with marked ICV in 3 of 7 cases [13], to mirror characteristics of oligoclonal expansions of normal LLPCs in the BM. The *IGHV* gene data on MGUS suggested origins from a GC B-cell exposed to on-going SHM, and that has acquired a neoplastic genomic Event 1 to by-pass the default apoptotic fate dependent on antigen selection pressure [13, 14]. Such a cell of origin in MGUS would be predicted to be committed transcriptionally to a PC fate, but able to undergo further rounds of SHM in the DZ. The inference from the MGUS findings is that a comparable normal GC B-cell may exist, committed to terminal maturation but able to re-enter the DZ and undergo SHM, possibly a ‘pre-PC’ [19].

In contrast to MGUS, in our and others' initial studies of MM-derived *IGHV* sequences, clonally homogeneous patterns of mutations were identified in individual MM cases, including in plateau phase of disease, but these data were based on analysis of limited numbers of clonally-related sequences [12]. The data suggested that neoplastic transformation occurs at a post-follicular stage in MM, with the cell of origin no longer exposed to SHM [12]. However, in our *IGHV* gene study of 2 matched pairs of MGUS that evolve to MM, we identified that a single clone evolves in each case, and observed that in 1 of 2 matched pairs variant *IGHV* tumor sequences persisted to the MM stage of disease [14]. These appeared to be derived from ‘MGUS-like’ cells in the MM clone [14].

**Figure 5:**
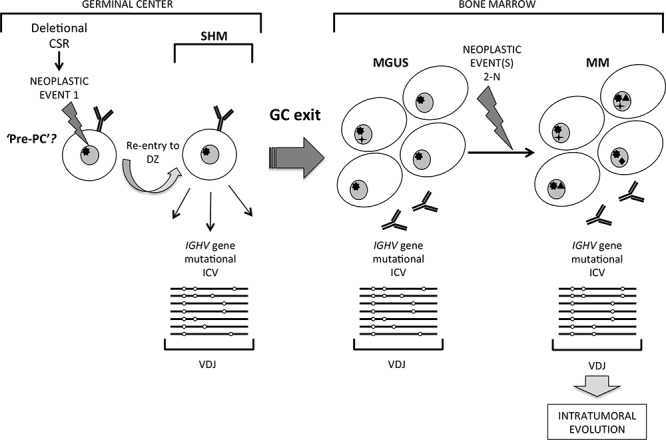
A distinct pathway of clonal evolution in MGUS and MM originating from a germinal center B cell undergoing somatic hypermutation Striking intraclonal variation (ICV) in patterns of tumor-derived *IGHV* gene sequence mutations in MM suggest a model in which a germinal center (GC) B cell that has undergone neoplastic Event 1, possibly at the stage of deletional class-switch recombination, re-enters the GC dark zone and is subjected to on-going somatic hypermutation (SHM). It is postulated that such a cell may be a ‘pre-PC’ committed to terminal maturation but able to undergo SHM, which exits the GC to home to the bone marrow to accumulate as MGUS. Subsequent sum of neoplastic Events 2-N in MGUS cells generate malignant MM, which retain the imprint of ICV resulting from the GC cell of origin being exposed to on-going SHM in tumor-derived *IGHV* genes.

Based on these initial observations, we hypothesized that clonal tumor-derived variants may be more common in symptomatic MM presentation disease that could be traced in *IGHV* genes with the enhanced sensitivity of deep next generation sequencing. In the present study, we indeed readily identified clonal *IGHV* gene sequences with ICV in 4 of 4 typical MM cases, substantiating our hypothesis that this may be a frequent feature of MM disease. This finding has only emerged from massive parallel sequencing of *IGHV* genes, and was not readily apparent from lower depth of analysis by conventional methods. These data are consistent with the concept that the cell of origin in these MM arises during the GC, from a B-cell continually targeted by SHM but committed to terminal maturation to generate the ICV we observe in clonal *IGHV* sequences, and mirrors origins of MGUS (Figure 5). Given the seminal observations that most if not all MM originate directly from MGUS [26, 27], our data suggest that MM with ICV in *IGHV* gene mutations arises from the same GC cell of origin as in MGUS with ICV in *IGHV* gene mutations, with disease presenting first as MGUS.

Although in 3 of 4 MM cases, clonal outgrowth is dominated by a single sequence subclone, the remaining fractions of variant sub-clonal sequences with ICV are nevertheless highly significant. This emerges when we examine the known MM tumor mass. In IgG MM the total tumor cells per patient has been calculated as 0.5 – 3.1 × 10^12^ based on data of *in-vivo* incorporation of ^125^I into IgG [28]. To illustrate this, in MM2 50.7% of the clonal reads are sub-clonal variant sequences with ICV over and above the dominant sequence. Taking the lower estimate of tumor mass, 50.7% of 0.5 × 10^12^ equates to 0.25 × 10^12^ transformed cells. Given that normal PCs only account for ~0.5–1.0% of mononucleated cells in the BM, and a total number of ~10^9^ comprising the complete polyclonal pool [29], the ‘variant’ tumor mass in MM1-4 can only be generated by proliferating transformed cells that persist.

Substantiating our observations, a recent study of a single MM case by massively parallel pyrosequencing also reported a dominant single clonal *IGHV* sequence with ICV [24]. Furthermore, in an initial report using the Lympho-SIGHT™ platform of high throughput sequencing of *IGHV* genes, in 401 MM cases, 71 (17.7%) showed evidence of clones related through SHM to the index clone at diagnosis [30]. Significantly, however, in the latter preliminary report [30], no conceptual framework was addressed to describe a possible pathway of clonal origins in MM in relation to the complexity of events in the GC, nor any association made with SHM patterns that we previously reported in MGUS [13]. Nevertheless, this initial report as an Abstract [30] appears to firmly substantiate our data that intraclonal variation in tumor-derived *IGHV* sequences occurs in MM. Additional high-throughput sequencing of *IGHV* genes in MM with the Lympho-SIGHT™ platform has also been reported directed at investigating minimal residual disease [15].

SHM levels in both MGUS and MM clonal *IGHV* genes are comparable in mutational load to normal LLPCs in the BM that largely derive from GC B-cells [11, 25], supporting the concept of GC origins of transformed PCs. MGUS cells retain expression of CD27 in most cases, although it is largely shed more frequently by aberrant MM PCs [31]. CD27 expression is acquired via GC trafficking [10].

With regard to a GC cell of origin that undergoes SHM and is committed to terminal maturation, previous investigations of IgD^+^ GC B-cells are noteworthy. In early studies, a subset of sIgM^−^IgD^+^CD38^+^ GC centroblasts were identified that have undergone class switch by Cμ–Cδ deletion, exhibit a marked λ light chain restriction and are supramutated in *IGHV* genes, but only differentiate to PCs *in-vivo* and do not circulate as memory B-cells [32]. Of high relevance, these IgD^+^ GC B-cells have also been proposed as the cell of origin in rare IgD^+^ MM disease, which reveal comparable genetic features of supramutated *IGHV* tumor-derived genes, a λ light chain bias and switch by Cμ deletion [32, 33].

Deletional CSR events occur in T-dependent antigen GC responses in B-cells [2, 4, 7]. Most MM are derived from isotype switched cells, and in MM that switch to IgG on the functional allele, a single switch event occurs that is retained and can be tracked as a signature nucleotide motif in relapse disease [34]. This is an important observation, as it shows clonal origins in MM from a *single* switch event that correctly ligates CSR on the functional allele. Such a switched cell must then re-enter the DZ to be subjected to further SHM to generate the ICV we observe in *IGHV* gene sequences in MM cells (schematically represented in Figure 5). The non-functional allele however provides important clues that the stage of deletional CSR events associates with the impact of Event 1 in clonal origins of MM. Dysregulated CSR activity on this allele generates chromosomal translocations that map to the *IgH* 14q32 locus in the main to S_H_ switch sites, and has been widely recognized as an early pathogenetic event in MM [35, 36]. Aberrant deletional CSR events most likely occur in a background of genomic instability that may have resulted from Event 1 (Figure 5), possibly of an epigenetic nature, but these as yet remain unidentified. Event 1 is also predicted to allow escape from the normal antigen affinity associated GC check-point to by-pass apoptosis. AID activity underpins aberrant CSR events, as evident in B-cell tumors characterized by *IgH* locus translocations [37]. Several 14q32 translocations have recurrent partner chromosomes, restricted to subsets but none are universal to MM disease [35]. It is conceivable that in MM lacking aberrant 14q32 chromosomal translocations deletional CSR progresses without being dysregulated, and that the nature of Event 1 may differ. In MGUS, a comparable spectrum of 14q32 chromosomal translocations also occur in disease [35], and may also associate with timing of Event 1 in the GC-derived cell of origin.

It is generally accepted that secondary event(s) or lesion(s) underlie malignant transformation of MGUS to MM. At present, it is unclear from our data whether the variant sequences identified in monoclonal MM *IGHV* transcripts are derived from residual ‘MGUS-like’ cells or whether these cells persist as subclonal expansions because they have independently acquired separate secondary hit(s) as Events 2-N (Figure 5). These events may include mutations in key genes that when estimated as % of the tumor population emerge as subclonal events in different MM patients: *KRAS* (20–72%), *NRAS* (32–96%), *BRAF* (36–92%) or *DIS3* genes (29–81%) [38]. Given that a long time-lag is usual in MGUS transforming to MM, subclonal neoplastic events could occur independently in cells with variant *IGHV* sequence. Data from mapping the genome in MM with aCGH and at the exome and whole-genome levels substantiate clonal evolution, where SNP abnormalities or acquired somatic mutations in individual tumors are shared only by some cells not others in individual cases, and on-going subclonal competition appears to promote tumor survival and progression [38–42]. Events 2-N in progression may also include epigenetic genome modifications or dysregulation at the miRNome level and microenvironment dependent modulation of tumor cells. Many mutations identified in MM cells already exist at the MGUS stage of disease [43, 44], and while many acquired somatic mutations are common to disease subsets none are universal, further closely associating the molecular pathways of origins in MGUS and MM.

The GC emerges as a crucible in genesis of MGUS and MM via a distinctive pathway in which the cell of origin is targeted by on-going SHM. The question now is what the nature(s) of Event 1 or additional lesions might be in GC origins of MGUS and MM. This pathway may also lead to MM in which clonal *IGHV* sequences are homogeneous even by deep sequencing, by a ‘cloning out’ phenomenon or that in clonally homogeneous MM the cell of origin may arise later with SHM silenced as we had proposed earlier [13, 14]. Fully elucidating these pathways will crystalize origins of MM, and inform models of disease [45, 46].

## MATERIALS and METHODS

### Patients and samples

Bone marrow samples of MM patients and normal donor (NPC1) were obtained for study with written informed consent in accordance with the Declaration of Helsinki and as approved by institutional ethics review committees. NPC1 was a donor undergoing orthopedic corrective surgery with no underlying disease. NPC2 cells were purchased from DV Biologics (Costa Mesa, CA, USA) as Bone Marrow Mononuclear Cells.

MM1-4 cases were previously untreated with symptomatic disease and CRAB features requiring active therapy (MM4 was plasma cell leukemia, with tumor cells purified from blood): MM1 IgGκ, MM2 IgGκ, MM3 IgGκ, and MM4 IgGλ. MM5 was an IgAκ tumor, previously treated and in 2^nd^ relapse and used as a control to assess any bias in deep sequencing IgG transcripts. MM1-4 samples were at diagnosis prior to therapy.

### PC purification

MM tumor cells were purified by automated CD138 magnetic cell sorting (Miltenyi). CD19^+^CD38^++^CD138^+^ normal PCs were purified by cell sorting using a BD FACSAria or FACSVantage (BD Biosciences, Oxford, UK) with CD19-APC-eFluor780/-CyChrome (eBioscience, Hatfield, UK; BD Biosciences), CD38-FITC (BioLegend, London, UK) and CD138-BV510/-PE (BD Biosciences).

### RNA

Total RNA was extracted from MM tumor cells and NPC using the Allprep or RNeasy kits (Qiagen, Crawley, UK) in accordance with the manufacturer's protocol.

### *IGHV* gene sequencing

Each RNA sample was separated into two and treated thereafter as two technical replicates.

cDNA was synthesized using SMARTscribe reverse transcriptase (Clontech Laboratories, Inc.) according to the manufacturer's recommended protocol. Briefly, 1 μL total RNA (1 to 145 ng) was added to 1 μL 20 μM 3′ SMART CDS Primer IIA and 2.5 μL dH_2_O and the sample was incubated at 72°C for 3 minutes, followed by 42°C for 2 minutes. Samples were supplemented with 2 μL 1st strand buffer, 0.25 μL 20 mM DTT, 1 μL 10 mM dNTPs, 1 μL SMARTer IIA oligonucleotide (5′ AAGCAGTGGTATCAACGCAGAGTACGCrGrGrG-3′), 0.25 μL recombinant RNase inhibitor and 1 μL Smartscribe reverse transcriptase, followed by incubation at 42°C for 1 hour.

*IGHV* region amplicons were generated from cDNA by PCR using individual pools of forward primers within framework region 1 (FR1) that were designed to amplify all known *IGHV* region alleles, and a reverse primer within the IgG constant region. Both primer sets incorporated Illumina P5 or P7 adaptor sequences at their 5′ ends to facilitate sequencing. We used Phusion Flash High-fidelity Taq polymerase (Life Technologies, UK) for PCR, which has a reported error rate of 4.4 × 10^−7^/bp/PCR cycle [47].

Amplicons were purified using an eGel Size-Select electrophoresis system (Life Technologies, UK) to select products within the anticipated size range of approximately 400–450 bp. 250 bp paired-end sequencing was performed on an Illumina MiSeq sequencer using a pool of read1 sequencing primers matching the pool of FR1 primers but omitting the Illumina adaptor sequence, an indexing primer to provide indexing information and a read2 primer matching the IgG constant region amplification primer that also lacked the Illumina adaptor sequence.

### Sequence quality control, paired end joining and filtering

Sequence read-pairs were combined using the Flash utility [48]. Sequence pairs which did not meet the quality criteria or which were shorter than 300 bp once combined were excluded from further analysis.

### Sequence analysis

All assembled variable region nucleotide sequences were processed using the VDJfasta utility [49]. VDJFasta uses a Hidden Markov Model to statistically analyze sequences upstream and downstream of putative CDR3s and outputs *V*, *D* and *J* germline sequences, CDR3 sequences and translated protein sequences derived from each read. To increase processing speed, sequence processing using this utility was parallelized using custom Perl and Python scripts and run on the parallel computing facility provided by the University of Edinburgh Compute and Data Facility (ECDF, http://www.ecdf.ed.ac.uk/).

### Neighbor-joining trees

For each donor, the 10 most prevalent variable region sequences derived from a clonal expansion with identical CDR3 were aligned using Clustal Omega, phylogenetic trees were calculated using the Neighbor-Joining algorithm [50] and trees rendered using FigTree v1.4 (http://tree.bio.ed.ac.uk/software/figtree/).

## SUPPLEMENTARY FIGURE AND TABLE



## References

[1] Lam KP, Kühn R, Rajewsky K (1997). *In vivo* ablation of surface immunoglobulin on mature B cells by inducible gene targeting results in rapid cell death. Cell.

[2] MacLennan IC (1994). Germinal centers. Annual Review of Immunology.

[3] Oracki SA, Walker JA, Hibbs ML, Corcoran LM, Tarlinton DM (2010). Plasma cell development and survival. Immunological Reviews.

[4] Gatto D, Brink R (2010). The germinal center reaction. Journal of Allergy and Clinical Immunology.

[5] Good-Jacobson KL, Shlomchik MJ (2010). Plasticity and heterogeneity in the generation of memory B cells and long-lived plasma cells: the influence of germinal center interactions and dynamics. The Journal of Immunology.

[6] Muramatsu M, Kinoshita K, Fagarasan S, Yamada S, Shinkai Y, Honjo T (2000). Class switch recombination and hypermutation require activation-induced cytidine deaminase (AID), a potential RNA editing enzyme. Cell.

[7] Tarlinton DM (2014). Immunology: To affinity and beyond. Nature.

[8] Allen CD, Okada T, Tang HL, Cyster JG (2007). Imaging of germinal center selection events during affinity maturation. Science.

[9] Toellner KM, Gulbranson-Judge A, Taylor DR, Sze DM, MacLennan IC (1996). Immunoglobulin switch transcript production *in vivo* related to the site and time of antigen-specific B cell activation. The Journal of Experimental Medicine.

[10] Klein U, Rajewsky K, Küppers R (1998). Human immunoglobulin (Ig)M+IgD+ peripheral blood B cells expressing the CD27 cell surface antigen carry somatically mutated variable region genes: CD27 as a general marker for somatically mutated (memory) B cells. The Journal of Experimental Medicine.

[11] Tarlinton D, Radbruch A, Hiepe F, Dorner T (2008). Plasma cell differentiation and survival. Current Opinion in Immunology.

[12] Stevenson F, Sahota S, Zhu D, Ottensmeier C, Chapman C, Oscier D, Hamblin T (1998). Insight into the origin and clonal history of B-cell tumours as revealed by analysis of immunoglobulin variable region genes. Immunological Reviews.

[13] Sahota SS, Leo R, Hamblin TJ, Stevenson FK (1996). Ig VH gene mutational patterns indicate different tumor cell status in human myeloma and monoclonal gammopathy of undetermined significance. Blood.

[14] Zojer N, Ludwig H, Fiegl M, Stevenson FK, Sahota SS (2003). Patterns of somatic mutations in VH genes reveal pathways of clonal transformation from MGUS to multiple myeloma. Blood.

[15] Martinez-Lopez J, Lahuerta JJ, Pepin F, González M, Barrio S, Ayala R, Puig N, Montalban MA, Paiva B, Weng L, Jiménez C, Sopena M, Moorhead M (2014). Prognostic value of deep sequencing method for minimal residual disease detection in multiple myeloma. Blood.

[16] Gitlin AD, Shulman Z, Nussenzweig MC (2014). Clonal selection in the germinal centre by regulated proliferation and hypermutation. Nature.

[17] Klein U, Casola S, Cattoretti G, Shen Q, Lia M, Mo T, Ludwig T, Rajewsky K, Dalla-Favera R (2006). Transcription factor IRF4 controls plasma cell differentiation and class-switch recombination. Nature Immunology.

[18] Sciammas R, Shaffer AL, Schatz JH, Zhao H, Staudt LM, Singh H (2006). Graded expression of interferon regulatory factor-4 coordinates isotype switching with plasma cell differentiation. Immunity.

[19] Kallies A, Hasbold J, Fairfax K, Pridans C, Emslie D, McKenzie BS, Lew AM, Corcoran LM, Hodgkin PD, Tarlinton DM, Nutt SL (2007). Initiation of plasma-cell differentiation is independent of the transcription factor Blimp-1. Immunity.

[20] Lanzavecchia A, Sallusto F (2002). Progressive differentiation and selection of the fittest in the immune response. Nature Reviews Immunology.

[21] Kelsoe G (1996). Life and death in germinal centers (redux). Immunity.

[22] Sungalee S, Mamessier E, Morgado E, Grégoire E, Brohawn PZ, Morehouse CA, Jouve N, Monvoisin C, Menard C, Debroas G, Faroudi M, Mechin V, Navarro JM (2014). Germinal center re-entries of BCL2-overexpressing B cells drive follicular lymphoma progression. Journal of Clinical Investigation.

[23] Smith KG, Light A, Nossal GJ, Tarlinton DM (1997). The extent of affinity maturation differs between the memory and antibody-forming cell compartments in the primary immune response. The EMBO Journal.

[24] Tschumper RC, Asmann YW, Hossain A, Huddleston PM, Wu X, Dispenzieri A, Eckloff BW, Jelinek DF (2012). Comprehensive assessment of potential multiple myeloma immunoglobulin heavy chain V-D-J intraclonal variation using massively parallel pyrosequencing. Oncotarget.

[25] Shlomchik MJ, Weisel F (2012). Germinal center selection and the development of memory B and plasma cells. Immunological Reviews.

[26] Landgren O, Kyle RA, Pfeiffer RM, Katzmann JA, Caporaso NE, Hayes RB, Dispenzieri A, Kumar S, Clark RJ, Baris D, Hoover R, Rajkumar SV (2009). Monoclonal gammopathy of undetermined significance (MGUS) consistently precedes multiple myeloma: a prospective study. Blood.

[27] Weiss BM, Abadie J, Verma P, Howard RS, Kuehl WM (2009). A monoclonal gammopathy precedes multiple myeloma in most patients. Blood.

[28] Salmon SE, Smith BA (1970). Immunoglobulin synthesis and total body tumor cell number in IgG multiple myeloma. J Clin Invest.

[29] Höfer T, Muehlinghaus G, Moser K, Yoshida T, E Mei H, Hebel K, Hauser A, Hoyer B, O Luger E, Dörner T, Manz RA, Hiepe F, Radbruch A (2006). Adaptation of humoral memory. Immunol Rev.

[30] Munshi NS, Minvielle S, Tai Y, Fulciniti M, Richardson PG, Attal M, Moreau P, Magranas F, Anderson KC, Faham M, Avet-Loiseau H (2014). Deep Sequencing of Immunoglobulin Loci Reveals Evolution of IgH Clone in Multiple Myeloma Patients over the Course of Treatment. (Abstract). Blood (ASH Annual Abstracts).

[31] Paiva B, Almeida J, Perez-Andres M, Mateo G, López A, Rasillo A, Vídriales MB, López-Berges MC, Miguel JF, Orfao A (2010). Utility of flow cytometry immunophenotyping in multiple myeloma and other clonal plasma cell-related disorders. Cytometry Part B. Clinical Cytometry.

[32] Arpin C, de Bouteiller O, Razanajaona D, Fugier-Vivier I, Brière F, Banchereau J, Lebecque S, Liu YJ (1998). The normal counterpart of IgD myeloma cells in germinal center displays extensively mutated IgVH gene, Cmu-Cdelta switch, and lambda light chain expression. Journal of Experimental Medicine.

[33] Arpin C, de Bouteiller O, Razanajaona D, Brière F, Banchereau J, Lebecque S, Liu YJ (1997). Human peripheral B cell development. sIgM-IgD+CD38+ hypermutated germinal center centroblasts preferentially express Ig lambda light chain and have undergone mu-to-delta switch. Annals of the New York Academy of Sciences.

[34] Taylor BJ, Kriangkum J, Pittman JA, Mant MJ, Reiman T, Belch AR, Pilarski LM (2008). Analysis of clonotypic switch junctions reveals multiple myeloma originates from a single class switch event with ongoing mutation in the isotype-switched progeny. Blood.

[35] Bergsagel PL, Kuehl WM (2001). Chromosome translocations in multiple myeloma. Oncogene.

[36] Sahota SS (2010). Modelling a role for IgH chromosomal translocations in the pathogenesis of B-cell tumours. Leukemia Research.

[37] Küppers R, Dalla-Favera R (2001). Mechanisms of chromosomal translocations in B cell lymphomas. Oncogene.

[38] Walker BA, Wardell CP, Melchor L, Hulkki S, Potter NE, Johnson DC, Fenwick K, Kozarewa I, Gonzalez D, Lord CJ, Ashworth A, Davies FE, Morgan GJ (2012). Intraclonal heterogeneity and distinct molecular mechanisms characterize the development of t(4,14) and t(11;14) myeloma. Blood.

[39] Bolli N, Avet-Loiseau H, Wedge DC, Van Loo P, Alexandrov LB, Martincorena I, Dawson KJ, Iorio F, Nik-Zainal S, Bignell GR, Hinton JW, Li Y, Tubio JM (2014). Heterogeneity of genomic evolution and mutational profiles in multiple myeloma. Nature Communications.

[40] Keats JJ, Chesi M, Egan JB, Garbitt VM, Palmer SE, Braggio E, Van Wier S, Blackburn PR, Baker AS, Dispenzieri A, Kumar S, Rajkumar SV, Carpten JD (2012). Clonal competition with alternating dominance in multiple myeloma. Blood.

[41] Egan JB, Shi CX, Tembe W, Christoforides A, Kurdoglu A, Sinari S, Middha S, Asmann Y, Schmidt J, Braggio E, Keats JJ, Fonseca R, Bergsagel PL (2012). Whole-genome sequencing of multiple myeloma from diagnosis to plasma cell leukemia reveals genomic initiating events, evolution, and clonal tides. Blood.

[42] Weston-Bell N, Gibson J, John M, Ennis S, Pfeifer S, Cezard T, Ludwig H, Collins A, Zojer N, Sahota SS (2013). Exome sequencing in tracking clonal evolution in multiple myeloma following therapy. Leukemia.

[43] Zhao S, Choi M, Heuck C, Mane S, Barlogie B, Lifton RP, Dhodapkar MV (2014). Serial exome analysis of disease progression in premalignant gammopathies. Leukemia.

[44] Walker BA, Wardell CP, Melchor L, Brioli A, Johnson DC, Kaiser MF, Mirabella F, Lopez-Corral L, Humphray S, Murray L, Ross M, Bentley D, Gutiérrez NC (2014). Intraclonal heterogeneity is a critical early event in the development of myeloma and precedes the development of clinical symptoms. Leukemia.

[45] Chesi M, Robbiani DF, Sebag M, Chng WJ, Affer M, Tiedemann R, Valdez R, Palmer SE, Haas SS, Stewart AK, Fonseca R, Kremer R, Cattoretti G (2008). AID-dependent activation of a MYC transgene induces multiple myeloma in a conditional mouse model of post-germinal center malignancies. Cancer Cell.

[46] Carrasco DR, Sukhdeo K, Protopopova M, Sinha R, Enos M, Carrasco DE, Zheng M, Mani M, Henderson J, Pinkus GS, Munshi N, Horner J, Ivanova EV (2007). The differentiation and stress response factor XBP-1 drives multiple myeloma pathogenesis. Cancer Cell.

[47] Kinde I, Wu J, Papadopoulos N, Kinzler KW, Vogelstein B (2011). Detection and quantification of rare mutations with massive parallel sequencing. Proceedings of the National Academy of Sciences U S A.

[48] Magoč T, Salzberg SL (2011). FLASH: fast length adjustment of short reads to improve genome assemblies. Bioinformatics.

[49] Glanville J, Zhai W, Berka J, Telman D, Huerta G, Mehta GR, Ni I, Mei L, Sundar PD, Day GM, Cox D, Rajpal A, Pons J (2009). Precise determination of the diversity of a combinatorial antibody library gives insight into the human immunoglobulin repertoire. Proceedings of the National Academy of Sciences U S A.

[50] Saitou N, Nei M (1987). The neighbor-joining method: a new method for reconstructing phylogenetic trees. Molecular Biology and Evolution.

