# Supplementary material for: Massive parallel IGHV gene sequencing reveals a germinal center pathway in origins of human multiple myeloma
Source: Oncotarget. 2015 Apr 10;6(15):13229–40. doi: 10.18632/oncotarget.3644 (PMC4537010; doi:10.18632/oncotarget.3644)
Supplement: Supplementary file 1 [file oncotarget-06-13229-s001.pdf]

SUPPLEMENTARY FIGURE AND TABLE

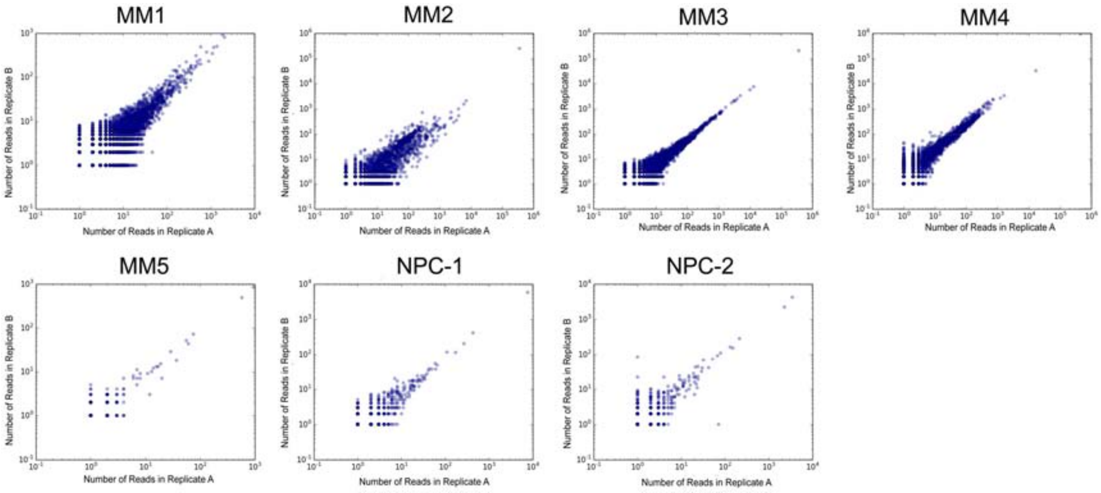

Supplementary Figure S1: Read counts for all QC-passed full-length *IGHV* gene variant sub-clone sequences that display an identical tumor-derived CDR3 sequence. The number of reads for each unique sequence in the two technical replicates A and B from each MM and NPC sample are aligned as a XY scatterplot, using a base 10 logarithmic scale.

Supplementary Table S1. Read counts in MM1-4 showing total reads and proportions of dominant sequence and remaining subclonal variant sequences

Case	Total reads	Top Read count	Sum variant reads count*	% Sum variant reads/total reads
MM1	1660375	2862	1657513	99.8
MM2	1232656	608012	624644	50.7
MM3	1218020	571958	646062	53.0
MM4	2009338	1393460	615878	30.7

*Sum variant reads counts is Total reads minus Top read count
